# Prediction of vaginal birth after cesarean delivery in Southeast China: a retrospective cohort study

**DOI:** 10.1186/s12884-020-03233-y

**Published:** 2020-09-15

**Authors:** Hua-Le Zhang, Liang-Hui Zheng, Li-Chun Cheng, Zhao-Dong Liu, Lu Yu, Qin Han, Geng-Yun Miao, Jian-Ying Yan

**Affiliations:** 1 Department of Obstetrics and Gynecology, Fujian Maternity and Child Health Hospital, Affiliated Hospital of Fujian Medical University, No.18, Daoshan Rd., Gulou Dist, Fuzhou City, Fujian province China; 2 Fujian Medical University, Fuzhou, China

**Keywords:** Mode of delivery decisions, Cesarean section, Vaginal birth, VBAC

## Abstract

**Background:**

We aimed to develop and validate a nomogram for effective prediction of vaginal birth after cesarean (VBAC) and guide future clinical application.

**Methods:**

We retrospectively analyzed data from hospitalized pregnant women who underwent trial of labor after cesarean (TOLAC), at the Fujian Provincial Maternity and Children’s Hospital, between October 2015 and October 2017. Briefly, we included singleton pregnant women, at a gestational age above 37 weeks who underwent a primary cesarean section, in the study. We then extracted their sociodemographic data and clinical characteristics, and randomly divided the samples into training and validation sets. We employed the least absolute shrinkage and selection operator (LASSO) regression to select variables and construct VBAC success rate in the training set. Thereafter, we validated the nomogram using the concordance index (C-index), decision curve analysis (DCA), and calibration curves. Finally, we adopted the Grobman’s model to perform comparisons with published VBAC prediction models.

**Results:**

Among the 708 pregnant women included according to inclusion criteria, 586 (82.77%) patients were successfully for VBAC. Multivariate logistic regression models revealed that maternal height (OR, 1.11; 95% CI, 1.04 to 1.19), maternal BMI at delivery (OR, 0.89; 95% CI, 0.79 to 1.00), fundal height (OR, 0.71; 95% CI, 0.58 to 0.88), cervix Bishop score (OR, 3.27; 95% CI, 2.49 to 4.45), maternal age at delivery (OR, 0.90; 95% CI, 0.82 to 0.98), gestational age (OR, 0.33; 95% CI, 0.17 to 0.62) and history of vaginal delivery (OR, 2.92; 95% CI, 1.42 to 6.48) were independently associated with successful VBAC. The constructed predictive model showed better discrimination than that from the Grobman’s model in the validation series (c-index 0.906 VS 0.694, respectively). On the other hand, decision curve analysis revealed that the new model had better clinical net benefits than the Grobman’s model.

**Conclusions:**

VBAC will aid in reducing the rate of cesarean sections in China. In clinical practice, the TOLAC prediction model will help improve VBAC’s success rate, owing to its contribution to reducing secondary cesarean section.

## Background

Reducing the rate of cesarean section is a global consensus. However, increase in cesarean delivery (CD) seems uncontrollable, with no signs of slowing down [1]. In China, statistical data indicate that CD rate has increased, by 20.8%, between 2008 and 2014, with this rate reportedly higher in certain regions [2]. Repeat cesarean delivery is the most important component, although implementation of the two-child policy has presented new challenges to reducing repeat cesarean delivery [3–5]. Since the 1970s, numerous studies have proposed the use of trial of labor after cesarean (TOLAC) as a strategy for reducing cesarean section rates, owing to the high success of VBAC ratio, coupled with associated low rates of adverse outcomes and cost effectiveness [6, 7]. Consequently, several professional organizations have tended to subject their patients to TOLAC. In fact, clinical practice guidelines support this evidence-based practice across such circumstances. In China’s tense doctor-patient relationships, clinical practice guidelines can be used at different levels of hospitals to support evidence-based practice during such circumstances. Among the existing clinical guidelines, Southeast China pays more attention to the 2015RCOG guidelines, with the subsequent release of Chinese guidelines also using these it as an important reference standard [8, 9]. However, TOLAC’s failure has been implicated in numerous perinatal risks, compared to elective repeated cesarean delivery without labor [10]. To avoid doctor-patient disputes, resulting from TOLAC failure, Chinese medical staff need an effective intervention. This calls for evaluation of TOLAC’s efficacy before and after delivery. To date, however, numerous studies have only reported computational and individualized risk assessment for successful TOLAC [11, 12]. Moreover, some prediction and validation models have been developed for pregnant Chinese women [13–15], although these models are based on local clinical guidelines and lack inclusion criteria for TOLAC. Generally, medical staff in southeastern China are more likely to practice TOLAC, based on admission criteria described clinical guidelines. Therefore, development of a prediction model based on this demand may help to improve TOLAC practice and reduce CD rates. The present retrospective study aimed to build a personalized prediction model for successful application of TOLAC in a population, based on admission criteria described in clinical guidelines. This model is expected to guide evaluation of VBAC feasibility before delivery.

## Methods

### Study design and data acquisition

This retrospective observational study was performed using data obtained from a case register, at the Fujian Provincial Maternity and Children’s Hospital A tertiary hospital in southeastern China between October 2015 and October 2017. This is a specialized hospital that serves nine prefecture-level cities, with an annual delivery volume of nearly 20,000 patients. Patient data, for this study, was extracted from medical records using pre-defined data fields.

### Selection criteria

Inclusion and exclusion criteria were assessed by investigators, according to China’s clinical guidelines for VBAC [9]. Summarily, inclusion criteria were as follows: women with a singleton pregnancy of cephalic presentation at 37 weeks or beyond, who have had a single previous lower segment caesarean delivery. The indications for the previous cesarean section did not appear again in this pregnancy before labour, such as abnormal fetal position, placenta previa, Oligohydramnios, severe preeclampsia, placental abruption, and twin pregnancy. In addition, all patients had an estimated fetal weight > 4000 g, and had an intact lower segment uterine scar following ultrasound analysis. On the other hand, women with two or more prior caesarean sections, a classical cesarean scar or previous uterine rupture, as well as those who have other absolute contraindications to vaginal birth, were excluded from the study. Moreover, all participants had no diabetes, chronic hypertension, cardiac disease, asthma, renal disease, or a connective tissue disorder. Finally, all participants experienced spontaneous uterine contractions and did not induce labor.

### Study outcomes

Primary outcomes were based on TOLAC’s success rate. On the other hand, secondary outcomes comprised analysis of maternal features, such as uterine rupture, mortality, and post-partum hemorrhage (estimated blood loss of more than 500 and 1000 ml for vaginal and cesarean deliveries, respectively), whereas neonatal outcome features included mortality, and neonatal asphyxia (defined as 5-min Apgar score ≤ 7).

### Predictors

Independent variables were extracted from medical record databases, based on values recorded at the time of development of spontaneous symptoms. Summarily, the extracted demographic information included age (on delivery date), maternal height, pre-gravid maternal weight, maternal weight at delivery, gravida, parity, abdominal, fundal height, cervix Bishop score (assessed after regular uterine contractions with abdominal pain for 2 h), history of vaginal delivery and gestational age.

### Statistical analysis

We used packages implemented in R software (V3.6.2) for all statistical analyses and generation of drawings, at a 95% significance interval. Comparisons between demographic and clinical characteristics were performed by outcome status, using t tests or χ^2^ tests. Construction and validation of the nomogram: To construct and validate the nomogram, we first incorporated clinical features as predictors, in its design. Summarily, 70 % (*n* = 483) of the participants were randomly assigned to the training cohort, whereas the rest of (*n* = 225) were assigned to the test cohort. We used the least absolute shrinkage and selection operator (LASSO) regression, with 5-fold cross-validation, to select the most useful predictive variables via 1se criteria for nomogram in the training cohort. In the test cohort, we first undertook internal validation, with a concordance index (C-index) estimation, then plotted calibration curves to determine concordance of the predicted and observed probabilities for survival time. Bootstrap resampling (1000 resamples) was used for this plot. Moreover, we evaluated clinical usefulness of the nomograms using decision curve analysis (DCA). Finally, we validated the Grobman’s model [12], in the test cohort, and compared it with our model.

### Ethical approval

This retrospective study was approved by the local institutional review boards and ethical committees. Written informed consent was not required because unidentifiable patient information used only.

## Results

### Sample characteristics of the cohort

**Fig. 1:**
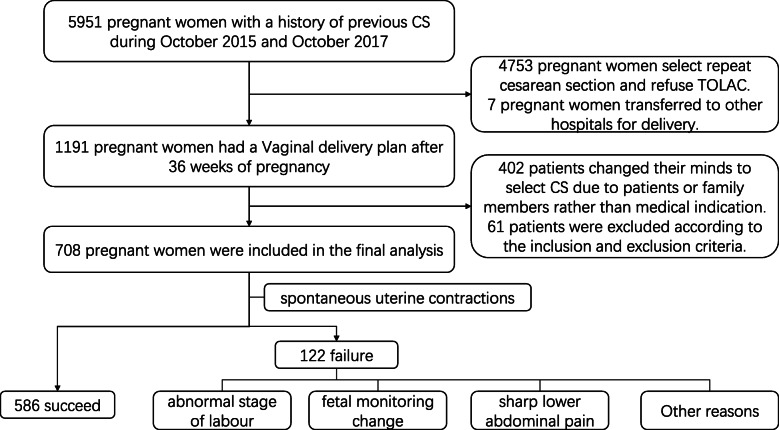
Flowchart of the enrolled patients

A total of 5951 pregnant women with a history of previous CS were identified during the observation period. One thousand one hundred ninety-one of these participants had a vaginal delivery plan, after 36 weeks of pregnancy, and these comprised the sample for the candidate. Participants who changed their minds, for caesarean section during vaginal delivery by choice or family member influence, rather than based on medical indication, were excluded from the study. Eventually, 708 pregnant women, all Asian, were included in the final analysis (Fig. 1). The indications of previous caesarean section mainly include the following categories: breech or shoulder presentation (19.4%), slow progress or arrest in labour (21.3), fetal distress (16.3%), without medical indication (12.4%), macrosomia (8.5%), oligohydramnios (6.9%), placenta previa (2.7%), twin pregnancy (2.4%), severe preeclampsia (1.6%), placental abruption (0.7%) and other indications. Clinical characteristics of the training (*n* = 483) and validation (*n* = 225) cohorts revealed no statistically significant differences between the groups (Table 1). Participants’ mean maternal age, at delivery, was 31.28 (SD: 3.64) years whereas their median gestational period was 39 weeks (IQR: 38.29–39.86). None of the pregnant women under this study experienced preeclampsia or gestational hypertension during pregnancy. The overall TOLAC success rate across the cohorts was 82.8%. In addition, 5.5% (39) of all women undergoing TOLAC were diagnosed with postpartum haemorrhage, 0.28% (2) had uterine rupture, with 0.14% (1) of new-borns experiencing neonatal asphyxia. In addition, no maternal death and hysterectomy were noted. TOLAC failure was attributed to abnormal stage of labour (34.4%), fetal monitoring change (33.6%), sharp lower abdominal pain (14.8%), fever or abnormal bleeding (9.8%) and other (7.4%).

**Table 1:** Clinical characteristics of the training and validation cohorts

Characteristics		Whole cohort (*n* = 708)	Training cohorts(*n* = 483)	Validation cohorts(*n* = 225)	P*
Maternal height (mean (SD))		1.60 (0.05)	159.80 (4.87)	159.27 (5.00)	0.180
Pre-gravid maternal weight (mean (SD))		53.27 (7.19)	53.43 (7.43)	52.93 (6.64)	0.383
Maternal weight at delivery (mean (SD))		66.74 (7.84)	67.07 (8.03)	66.02 (7.37)	0.095
Pre-gravid maternal BMI (median [IQR])		20.62 [19.15, 22.42]	20.57 [19.07, 22.48]	20.78 [19.33, 22.06]	0.703
Maternal BMI at delivery (median [IQR])		26.16 [24.36, 27.88]	26.20 [24.34, 27.96]	26.02 [24.50, 27.50]	0.392
Abdominal circumference (mean (SD))		98.37 (5.05)	98.44 (5.23)	98.21 (4.62)	0.582
Fundal height (mean (SD))		33.87 (1.50)	33.91 (1.57)	33.78 (1.31)	0.253
Cervix Bishop score (median [IQR])		7.00 [7.00, 8.00]	7.00 [7.00, 8.00]	7.00 [7.00, 8.00]	0.909
Maternal age at delivery (mean (SD))		31.28 (3.64)	31.23 (3.58)	31.39 (3.78)	0.574
Gestation (median [IQR])		39.00 [38.29, 39.86]	1.00 [0.00, 1.00]	1.00 [0.00, 1.00]	0.334
Cesarean section interval time (median [IQR])		5.00 [3.00, 7.00]	5.00 [3.00, 6.50]	4.00 [3.00, 7.00]	0.942
History of vaginal delivery (%)	NO	473 (66.8)	319 (66.0)	154 (68.4)	0.585
	YES	235 (33.2)	164 (34.0)	71 (31.6)	
Rupture of membranes (%)	NO	473 (66.8)	332 (68.7)	141 (62.7)	0.131
	YES	235 (33.2)	151 (31.3)	84 (37.3)	
Success of TOLAC (%)	NO	122 (17.2)	86 (17.8)	36 (16.0)	0.627
	YES	586 (82.8)	397 (82.2)	189 (84.0)	

*t test or χ^2^ test; Mann-Whitney U test was applied for Non-normally distributed data

*IQR* interquartile range, *BMI* Body Mass Index, *SD* standard deviation

### Predictors of TOLAC success

**Fig. 2:**
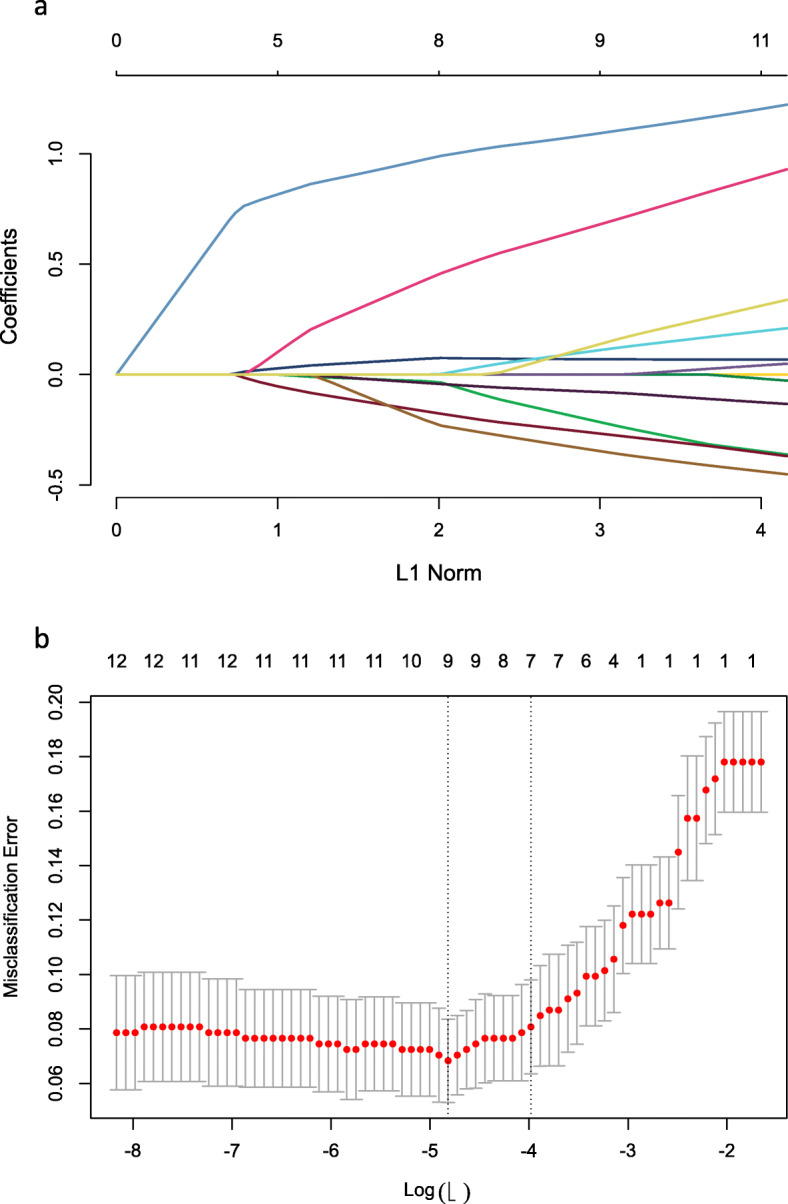
Feature selection using the least absolute shrinkage and selection operator (LASSO) logistic regression model. **a** LASSO coefficient profiles of the 13 features describing success rate of TOLAC. **b** Tuning parameter (lamda) selection in the LASSO model used 5-fold cross-validation via minimum criteria for determining success rate of TOLAC

**Fig. 3:**
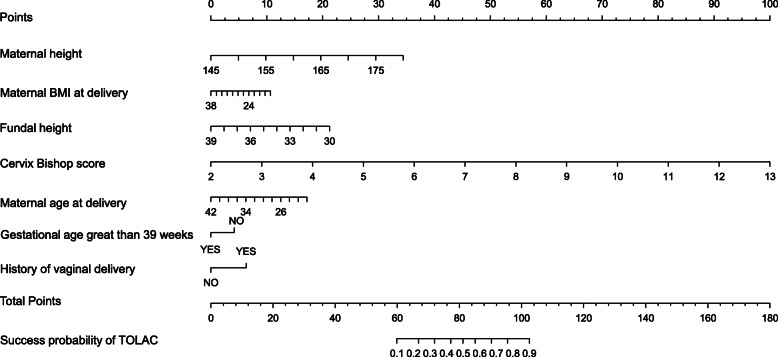
Nomogram for predicting success rate of TOLAC

Univariate analysis revealed that maternal height, maternal BMI, parity, fundal height, cervix bishop score, duration of labour, maternal age, history of vaginal delivery and rupture of membranes were associated with successful TOLAC (Table 2). During nomogram development, we incorporated clinical characteristics as prognostic features, with all these parameters reduced to the most useful potential predictors during determination of the TOLAC’s success rate in the training cohort, using the LASSO logistic regression model. Consequently, results from the LASSO logistic regression model were incorporated into the nomogram and used to predict success rate of TOLAC (Fig. 2). Summarily, maternal height, and BMI at delivery, fundal height, cervix Bishop score, maternal age at delivery, gestation period greater than 39 weeks and history of vaginal delivery were independent predictors for TOLAC (Fig. 3 and Table 3).

**Table 2:** Sample characteristics based on TOLAC status

Characteristics		Failure of TOLAC(*n* = 122)	Success of TOLAC(*n* = 586)	P*
Maternal height (mean (SD))		1.58 (0.05)	1.60 (0.05)	< 0.001
Pre-gravid maternal weight (mean (SD))		53.55 (7.56)	53.21 (7.11)	0.635
Maternal weight at delivery (mean (SD))		67.64 (7.98)	66.55 (7.80)	0.164
Pre-gravid maternal BMI (median [IQR])		21.23 [19.71, 22.95]	20.57 [19.04, 22.26]	0.013
Maternal BMI at delivery (median [IQR])		27.10 [25.24, 28.78]	26.00 [24.20, 27.60]	< 0.001
Parity (median [IQR])		1.00 [1.00, 2.00]	1.00 [1.00, 3.00]	< 0.001
Abdominal circumference (mean (SD))		98.56 (5.26)	98.33 (5.00)	0.639
Fundal height (mean (SD))		34.32 (1.60)	33.78 (1.46)	< 0.001
Cervix Bishop score (median [IQR])		6.00 [4.00, 6.00]	8.00 [7.00, 8.00]	< 0.001
Duration time of labor (median [IQR])		7.00 [4.00, 10.75]	5.57 [4.10, 8.30]	0.044
Maternal age at delivery (mean (SD))		31.87 (3.57)	31.16 (3.64)	0.049
Gestation (median [IQR])		39.00 [38.00, 40.00]	39.00 [38.29, 39.86]	0.177
Cesarean section interval time (median [IQR])		5.00 [3.00, 7.00]	5.00 [3.00, 7.00]	0.28
History of vaginal delivery (%)	NO	104 (85.2)	369 (63.0)	< 0.001
	YES	18 (14.8)	217 (37.0)	
PROM (%)	NO	96 (78.7)	377 (64.3)	0.003
	YES	26 (21.3)	209 (35.7)	

*t test or χ^2^ test; Mann-Whitney U test was applied for Non-normally distributed data

**Table 3:** Predictors of TOLAC success rate based on the nomogram

	β	SE	OR [95%CI]	*P*
(Intercept)	−5.29723	6.280949	0.01 [0.00, 1041.20]	0.399
Maternal height	0.105889	0.035648	1.11 [1.04, 1.19]	0.003
Maternal BMI at delivery	−0.11339	0.058858	0.89 [0.79, 1.00]	0.054
Fundal height	−0.33551	0.108055	0.71 [0.58, 0.88]	0.002
Cervix Bishop score	1.183312	0.147888	3.27 [2.49, 4.45]	< 0.001
Maternal age at delivery	−0.10831	0.044441	0.90 [0.82, 0.98]	0.015
Gestational age great than 39 weeks	−1.09738	0.323738	0.33 [0.17, 0.62]	0.001
History of vaginal delivery	1.070891	0.385452	2.92 [1.42, 6.48]	0.005

*OR* odds ratio, *CI* confidence interval

### Nomogram validation and compare

**Fig. 4:**
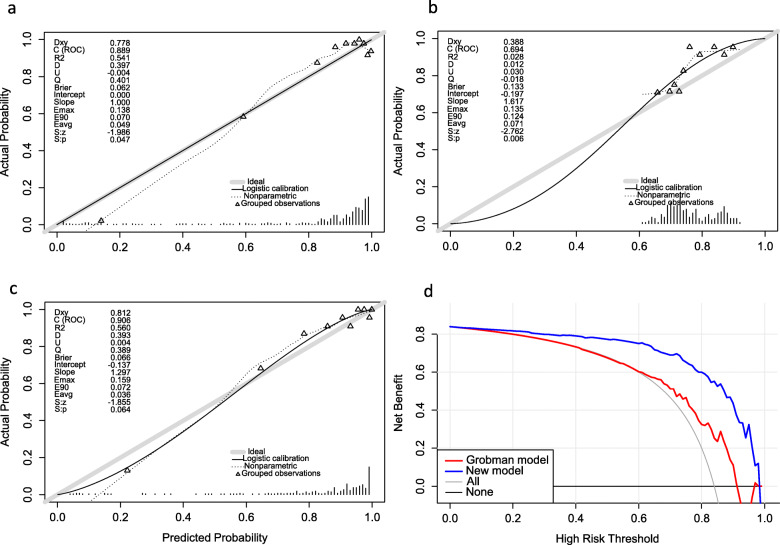
Calibration curves of the nomogram in training cohort and validation cohorts of TOLAC. **a** Prediction of success rate of TOLAC in training cohort of TOLAC. **b** Prediction of success rate of TOLAC using the Grobman’s model for validation (test) cohort of TOLAC. **c** Calibration curves for predicting success of TOLAC nomogram construction (Bootstrap = 1000 repetitions) in validation (test) cohort of TOLAC. **d**. Decision curve analysis for two method

Predictive accuracy, for the success rate of TOLAC as measured by C-index was 0.89 in the internal validation. The calibration plot for the probability of TOLAC’s success showed a strong correlation between the actual (observed) outcome and that predicted by the nomogram (Fig. 4a). In addition, we plotted calibration curves to evaluate performance of the newly-developed nomogram and Grobman’s model in the test cohort, respectively (Fig. 4b and c). Results indicated that the newly-developed nomogram model was superior, to the Grobman’s model, in predicting patients for inclusion in the standard according to clinical guidelines, based on data in Fig. 3. This was evidenced by both correlation and c-index (c-index: 0.90 vs 0.69, respectively).

### Decision curve analysis

Decision curves for newly-developed nomogram and Grobman’s models, used to predict the rate of TOLAC in patients are illustrated in Fig. 4d. Here, the new model was useful, with threshold probabilities of 60–90%. In addition, the calibration curves revealed a higher success rate in actual vaginal delivery in this interval.

## Discussion

Previous studies have described the importance of adopting TOLAC for reduction of CD rates and improvement of maternal and child outcomes [6]. In fact, the Generalizing Grobman’s model has been found to successfully predict the rate of TOLAC in Chinese populations, with a strong clinical predictive power [13, 15]. However, sample populations included in these studies have lacked uniform standards. Given the numerous efforts in clinical guidelines for obstetrics in China, in recent years, aimed at reducing medical disputes and improving the quality of healthcare, it is important to ensure accurate selection of patients to be predicted based on clinical guidelines. This is because prediction models are only applied to single patient populations selected based on similar inclusion and exclusion criteria as well as clinical management [16]. Since the rate of TOLAC in China is very low, establishment of a reliable predictive model in low-risk pregnant women, without serious complications, is imperative to improving the TOLAC rate without too much medical risk. The present study aimed to identify factors that influence the success rate of TOLAC, and develop a predictive model to guide effective implementation of clinical guidelines. Particularly, we used external verification of the widely used Grobman’s model, to add corresponding features before delivery, and achieved superior predictive ability.

Based on a clear trend of benefits from the successful vaginal delivery among those trials of labor after cesarean section, it is evident that VBAC failure exacerbates many risks, including bleeding, increased blood transfusions, uterine rupture and endometritis, as well as infant asphyxia or perinatal death [8, 17, 18]. Consequently, obstetricians prefer a more conservative approach during TOLAC to avoid medical disputes owing to the complex physician-patient relationship as well as the associated high work-related stress. When longer labor course or changes in fetal heart rate occur during TOLAC, doctors are more likely to perform repeat cesarean sections, in order to avoid the associated adverse consequences of uterine rupture or neonatal asphyxia. In the present study, our results indicate that the incidence of adverse clinical outcomes, such as uterine rupture and neonatal asphyxia, are lower than what has previously been reported. However, this strategy also significantly reduces TOLAC’s success rate. In the present study, the described risk model had several advantages over the Grobman’s model, with regards to discrimination and calibration. For example, it increased predictive selection power near delivery.

The inclusion and exclusion criteria, employed in this study, were based on recent clinical guidelines, whereas the pre-delivery variables were increased by modifying the Grobman’s model. These results are consistent with recent studies reporting that maternal BMI at delivery, history of vaginal delivery and maternal age at delivery are relevant or independent risk factors for successful TOLAC [12, 19–22]. Among these factors, history of vaginal delivery for predicting TOLAC success has been extensively reported [23]. In fact, maternal age is correlated with the success of TOLAC, despite the increase in the proportion of older pregnant women being affected by China’s recent two-child policy. Given the previously reported differences in BMI, between different races [24], maternal BMI was a continuous variable in the model. In addition, results from LASSO screening indicated that maternal pre-pregnancy weight is not an independent risk factor for TOLAC’s success, whereas maternal weight at delivery was associated with the success rate.

Bishop’s score is a relatively subjective indicator for standardization. In the present study, we looked up the cervical bishop’s score, two hours after regular uterine contractions, and simultaneously analyzed results from midwives and obstetricians. We found a positive correlation between Bishop’s score and success of TOLAC, consistent with Francis (2005) who demonstrated a relationship between risk of cesarean delivery and unfavorable Bishop score at admission [25]. Similarly, several related studies have confirmed that the Bishop’s score, at delivery, affects the success rate of TOLAC [17, 26]. In the present study, the OR of Bishop’s score was higher, with a relatively narrower confidence interval than that reported in previous studies (OR, 3.27; 95% CI, 2.49 to 4.55). It is possible that previously reported models may have underestimated the role of standardized cervical evaluation.

Generally, previous studies have shown that maternal pelvis shape and fetal weight are the determining factors for the success of TOLAC [13, 21]. However, estimating both parameters is challenging. In addition, a strong relationship has been reported between maternal pelvis shape and their height [27]. Results from the present study showed that higher pregnant women had a bigger chance of TOLAC success, suggesting that maternal height could be an independent factor for successful TOLAC. Determination of fetal weight by ultrasound scan results in low accuracy, and is also easily affected by the experience of the personnel performing it [28]. In addition, ultrasound scans are difficult to standardize the estimated weight, between different hospitals. Consequently, we chose the fundal height and maternal abdominal as the indicators for inclusion into the model, and found that fundal height was negatively associated with TOLAC success.

Previous studies have proposed the use of pregnancy at 40 weeks as a cut-off point for developing prediction models [29, 30]. In the present study, we selected 39 weeks for delivered gestational weeks as a reference, based on the clinical guidelines. Similar results were observed using different cut-off values, with spontaneous uterine contractions before 39 gestational weeks found to be more conducive to successful delivery.

In this study, we did not evaluate performance on the hysterotomy scar, despite previous studies implicating it in prediction of TOLAC success [31]. This is because the examination is difficult to standardize, and is not conducive to further promote primary hospital, owing to the differences in experience of ultrasound practitioners as well as the associated examination methods. Recent evidence also suggests that models, based on the sonographic assessment of a hysterotomy scar, have poor accuracy in predicting successful VBAC [32].

This study had several limitations. Particularly, the study adopted a retrospective design, to recall of medical services received. Although we screened all vaginal trial cases, during the study period, some patients refused TOLAC and preferred repeat cesareans. In addition, a limited sample size presented a limitation to screening of clinical factors. Future studies are expected to include a bigger sample size to improve accuracy. Finally, we performed a single center analysis, targeting a population from Southeast China. Future studies are expected to include more populations across China and the world.

## Conclusions

We successfully developed a model for predicting the success rate of TOLAC in Southeast China, to circumvent the current low ratio (20%). The key strengths of this study are its inclusion and exclusion criteria, which were consistent with clinical guidelines. Our data also confirm the safety of TOLAC, in accordance with clinical guidelines. Overall, these findings are expected to guide obstetricians in southeast China to accurately predict TOLAC’s success rate, and have a number of practical implications. However, generalization of the model is proposed to further validate and broaden its applications. Further prospective research and multicenter clinical trials are needed to validate and refine the model.

## Data Availability

Data were anonymized, and no patient information was included to preserve confidentiality. All data used to reach the aforementioned conclusions is available for scientific purposes, if needed.

## Abbreviations

TOLAC: Trial of Labor After Cesarean
VBAC: Vaginal Birth After Cesarean;
BMI: Body Mass Index
CS: Caesarean Section
CD: Cesarean Delivery
IQR: Interquartile range
SD: Standard deviation
LASSO: Least Absolute Shrinkage and Selection Operator
OR: Odds Ratio
CI: Confidence interval
